# Genetics of the Human Interferon Lambda Region

**DOI:** 10.1089/jir.2019.0043

**Published:** 2019-09-27

**Authors:** Ludmila Prokunina-Olsson

**Affiliations:** Division of Cancer Epidemiology and Genetics, Laboratory of Translational Genomics, National Cancer Institute, National Institutes of Health, Bethesda, Maryland.

**Keywords:** type-III interferon, interferon lambda, *IFNL4*, IFN-λ4, GWAS, HCV, viral clearance

## Abstract

Humans are polymorphic in their ability to produce type-III interferons. Most individuals of African ancestry are genetically capable of generating all 4 type-III interferons (IFN-λ1, 2, 3, and 4), whereas the majority of individuals of European and Asian ancestry lack IFN-λ4 and thus can generate only IFN-λ1, 2, and 3. All 4 type-III IFNs are encoded by genes located within a ∼55 kb genomic region on human chromosome 19. Although IFN-λ4 appears to be important in animals, genetic alterations acquired in the Hominidae lineage, and particularly in humans, resulted in the elimination of IFN-λ4 or restriction of its activity, suggesting that IFN-λ4 function might be detrimental to human health. Genetic variants within the *IFNL* region, including those controlling production and activity of IFN-λ4, have been strongly associated with clearance of hepatitis C virus (HCV) infection. There is growing evidence for association of the same genetic variants with a multitude of other disease conditions. This article reviews the genetic landscape of the human *IFNL* genetic locus, with an emphasis on the genetic control of IFN-λ4 production and activity, and its association with viral clearance.

## Introduction

The *IFNL* locus has attracted considerable attention due to the strong association of genetic variants in this region with spontaneous and treatment-induced clearance of hepatitis C virus (HCV) infection. In 2009, several genome-wide association studies (GWASs) reported significant associations with HCV clearance for 2 single nucleotide polymorphisms (SNPs)–rs12979860 (Ge and others 2009; Thomas and others 2009) and rs8099917 (Suppiah and others 2009; Tanaka and others 2009). The difference between these results was primarily technical and dependent on the genotyping platform used. The 2 studies that reported rs8099917 as the strongest GWAS signal used genotyping platforms that did not include rs12979860—an Affymetrix 6.0 chip (Tanaka and others 2009) or an Illumina Infinium HumanHap300 chip (Suppiah and others 2009). In contrast, a study that used Illumina Human 610-Quad chip, which included both SNPs, reported a stronger association for rs12979860 than for rs8099917 in Europeans and Hispanics, whereas only rs12979860 was associated in African Americans (Ge and others 2009). Because rs12979860 was associated with viral clearance in all population groups tested, it became widely used and known as the representative “*IL28B* marker.”

Association of GWAS markers does not assume their functionality. However, these markers may be linked to many other variants in the same genomic region, and one or several of those genotyped, not genotyped, or even currently unknown variants may be responsible for relevant molecular phenotypes. Therefore, the discoveries made a decade ago (Ge and others 2009; Suppiah and others 2009; Tanaka and others 2009; Thomas and others 2009) started a quest to identify the causal genetic variants responsible for the GWAS signals detected for rs12979860 and rs8099917, as well as to understand how these variants could be affecting viral clearance. This article discusses the genetic landscape of the *IFNL* region and shows how genetic analysis of this region is helping to identify the molecular mechanism of HCV clearance and several additional disease conditions that have been associated with this genetic region.

### Physical versus genetic definition of the *IFNL* region

All 4 IFN-λs—IFN-λ1, IFN-λ2, and IFN-λ3 (formerly known as IL29, IL28A, and IL29, respectively (Kotenko and others 2003; Sheppard and others 2003), and the recently discovered IFN-λ4 (Prokunina-Olsson and others 2013)—are encoded within a ∼55 kb genomic region at the 19q13.2 cytoband on human chromosome 19. However, the genetic size of the *IFNL* region is much smaller than its physical size due to population-specific recombination events that broke this 55 kb genomic region into several linkage disequilibrium (LD) blocks of genetically linked markers. LD blocks are defined based on pair-wise correlations (*r*^2^) between markers, with an arbitrary threshold of *r*^2^ = 0.8 (80% correlation) being used to identify markers in high LD; these linked markers are expected to demonstrate comparable genetic associations.

**FIG. 1.**
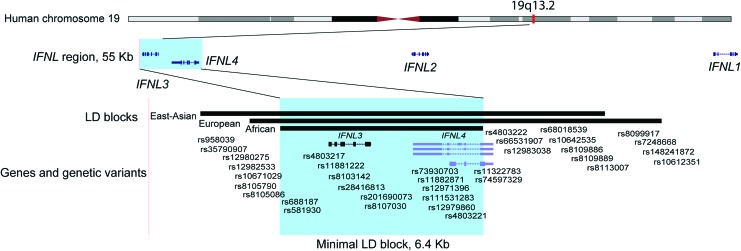
Physical and genetic map of the human *IFNL* region on chromosome 19q13.2. The plot is customized based on the map from the UCSC Browser (https://genome.ucsc.edu) (human genome reference GRCh37/hg19). LD blocks are generated based on information from the 1000 Genomes Project and include genetic variants in *r*^2^ > 0.8 with rs12979860 (Table 1). The *highlighted area* includes the overlap between LD blocks in populations of East Asian, European, and African ancestry. Populations of East Asian ancestry (EAS) include Han Chinese in Beijing, China (CHB), Japanese in Tokyo, Japan (JPT), Southern Han Chinese, China (CHS), Chinese Dai in Xishuanagbanna, China (CDX), and Kinh in Ho Chi Minh City, Vietnam (KVH). Populations of European ancestry (EUR) include Utah Residents (CEPH) with northern and western European ancestry (CEU), Toscani in Italy (TSI), Finnish in Finland (FIN), British in England and Scotland (GBR), and Iberian population in Spain (IBS). Populations of African ancestry (AFR) include Yoruba in Ibadan, Nigeria (YRI), Luhya in Webuye, Kenya (LWK), Mandinka in The Gambia (MAG), Mende in Sierra Leone (MSL), Esan in Nigeria (ESN), Americans of African Ancestry in SW USA (ASW), and African Caribbean in Barbados (ACB). LD Link tool (Machiela and Chanock 2015) was used to mine the 1000 Genomes Project data. LD, linkage disequilibrium. Color images are available online.

The 1000 Genomes Project (Genomes Project and others 2015) includes genotype data for 84.4 million genetic variants sequenced in 2,504 individuals from 26 populations, with 5–7 populations representing groups of East Asian, European, and African ancestry. Analysis of the 1000 Genomes Project data showed that rs12979860 LD block includes 33 SNPs within a 13,023 bp DNA fragment in East Asian populations, 22 SNPs within a 12,802 bp DNA fragment in populations of European ancestry, but only 8 SNPs within a 6,403 bp DNA fragment in populations of African ancestry (http://www.internationalgenome.org/1000-genomes-browsers; Fig. 1 and Table 1). Interestingly, rs8099917 is included in this block only in East Asians (*r*^2^ = 0.92 with rs12979860) but not in Europeans (*r*^2^ = 0.43) or Africans (*r*^2^ = 0.02). If rs12979860 represents the same genetic signal in individuals of all ancestries, then the 6.4 kb LD block of African ancestry is the common denominator between these population-specific blocks. This shared LD block includes the entire *IFNL3* and most of *IFNL4* but excludes variants in the *IFNL2* and *IFNL1* genes as irrelevant for this GWAS association.

**Table 1.** Genetic Markers Included in Linkage Disequilibrium Blocks, Defined by *r*^2^ > 0.8 with Genome-Wide Association Studies-Associated Single Nucleotide Polymorphism rs12979860

			*Ancestry, 1000 Genomes Projects populations, r^2^ with rs12979860*			
*SNP*	*Alleles*	*Position GRCh37/hg19*	*East Asian,* N* = 504*	*European* N* = 503*	*African* N* = 661*	*Gene*
rs958039	T/A	39730301		0.89		
rs35790907	A/T	39730755		0.83		
rs12980275	A/G	39731783	0.87	0.85		
rs12982533	T/C	39731904	0.83	0.86		
rs10671029	−/AA	39732197	0.80			
rs8105790	T/C	39732501	0.88			
rs8105086	A/G	39732712	0.97	0.94		
rs688187	G/A	39732752	0.97	0.95	0.94	*IFNL3*
rs581930	C/T	39733123	0.88	0.88		
rs4803217	C/A	39734220	0.97	0.98	0.88	*IFNL3*
rs11881222	A/G	39734923	0.95	0.91		
rs8103142	T/C	39735106	0.96	0.99		
rs28416813	C/G	39735644	0.97	0.96	0.89	*IFNL3*
rs201690073	C/T	39735919	0.97	0.90		
rs8107030	A/G	39736719	0.89			
rs73930703	C/T	39737513	1.0	0.99	0.94	*IFNL4*
rs11882871	A/G	39737610	1.0	0.98	0.86	*IFNL4*
rs12971396	C/G	39737866	0.93			
rs111531283	A/C	39738317	1.0	0.92		
rs12979860	C/T	39738787	1.0	1.0	1.0	*IFNL4*
rs4803221	C/G	39739129	0.93			
rs11322783	T/−	39739153	0.99	0.98	0.83	*IFNL4*
rs74597329	T/G	39739155	1.0	0.98	0.83	*IFNL4*
rs4803222	G/C	39739353	1.0	0.90		
rs66531907	C/A	39740675	0.93			
rs12983038	G/A	39741124	0.93			
rs68018539	C/T	39741465	1.0	0.89		
rs10642535	−/TC	39742683	0.99	0.95		
rs8109886	C/A	39742762	0.90			
rs8109889	C/T	39742770	0.92			
rs8113007	A/T	39743103	0.99	0.89		
rs8099917	T/G	39743165	0.92			
rs7248668	G/A	39743821	0.92			
rs148241872	−/GA	39744102	0.92			
rs10612351	AC/−	39744806	0.93			
LD block size			13,023 bp	12,802 bp	6,403 bp	

Shaded—markers included in LD blocks in corresponding population groups.

1000 Genomes Project populations are described in Fig. 1.

LD, linkage disequilibrium; SNP, single nucleotide polymorphism.

### *IFNL4* variants in high LD with rs12979860

Because the LD block shared in all populations includes only 8 markers—rs12979860 and 7 markers in high LD with it (*r*^2^ > 0.8, Table 1)—one or several of these variants are likely to be genetically and functionally responsible for this GWAS signal. Five of these markers are located within *IFNL4*. The GWAS marker rs12679860 is located within the first intron of *IFNL4,* and its functional significance is unclear. SNPs rs73930703 and rs11882871 are located within the 3′UTR of *IFNL4* and might affect the stability of this transcript. The other 2 *IFNL4* variants—rs11322783 (−/T) and rs74597329 (T/G)—are located within exon 1 of *IFNL4*. Because alleles of these SNPs always segregate together, they form a dinucleotide polymorphism, rs368234815, with the combined alleles TT/−G (also known as TT/dG or TT/ΔG). Initially, this polymorphism had been reported with a preliminary accession number ss469415590 (Prokunina-Olsson and others 2013), which was later replaced by a final dbSNP accession number rs368234815. In the Genotype-Tissue Expression (GTEx) project (Consortium 2013) and other studies that use the 1000 Genomes Project data, rs368234815 is represented by SNPs rs11322783 and rs74597329.

Both rs368234815 and rs12979860 show comparable association with viral clearance in populations with high LD between these markers (Asians and Europeans, Table 1). The analysis is more informative in African Americans, where association for rs368234815 is significantly stronger than for rs12979860 due to lower LD between these markers (Prokunina-Olsson and others 2013; Aka and others 2014). Owing to its location within the first exon of *IFNL4*, rs368234815 has a significant functional effect—the dG allele creates an open reading frame for the IFN-λ4 protein, whereas the TT allele introduces frame shift that abrogates IFN-λ4 by a premature stop codon (Prokunina-Olsson and others 2013).

The dG allele is invariably present in *IFNL4* genes of all animals. The 2-base change of dG to TT occurred in humans ∼60,000 years ago, just before the out-of-Africa migration (Key and others 2014). Interestingly, the whole region that includes the *IFNL3* and *IFNL4* genes is missing in the genomes of mice and rats, but the IFN-λ4 protein sequence is preserved by purifying selection in other animals, indicating its importance (Key and others 2014). Strong positive selection for the derived TT allele in many populations indicates that the loss of IFN-λ4 was beneficial, although the conditions that favored this selection are unclear (Key and others 2014).

Biology of IFN-λ4 is actively studied by many groups (Lu and others 2015a; Onabajo and others 2015; Fan and others 2016; Hong and others 2016; Obajemu and others 2017; Sung and others 2017). Current understanding of functional properties of all type-III IFNs, including IFN-λ4, has been reviewed elsewhere (O'Brien and others 2014; Wack and others 2015; Andreakos and others 2017; Hemann and others 2017; Wells and Coyne 2018) and will not be discussed in this article.

### Other functional *IFNL4* variants

*IFNL4* has 3 common nonsynonymous SNPs—rs73555604 (C17T) in exon 1 and rs142981501 (R60P) and rs117648444 (P70S) in exon 2 (Prokunina-Olsson and others 2013). Notably, all these nonsynonymous variants have emerged on the background of distinct haplotypes that encode IFN-λ4 (Table 2), and the emergence of these SNPs may represent an evolutionary mechanism to modulate IFN-λ4 activity. R60P exists only at a low (∼4%) allele frequency in individuals of African ancestry but is not found elsewhere, and C17Y frequency is high in African ancestry (26%) but is very low in other groups, with only ∼2% in Europeans and 0% in East Asians. The location of R60P next to N61, the unique glycosylation site, and C17Y within the leader peptide might be functionally important for IFN-λ4 activity, but these mechanisms remain to be explored.

**Table 2.** Haplotypes of Protein-Coding *IFNL4* Markers

			*Ex 1*									
	*Ex 2*	*Ex 2*	*rs368234815*		*Ex 1*		*Ancestry, 1000 Genomes Project populations*					
	*P70S*	*R60P*	*IFN-λ4/no IFN-λ4*		*C17Y*	*Outcome*	*East Asian*		*European*		*African*	
*Haplotypes*	*rs117648444*	*rs142981501*	*rs11322783*	*rs74597329*	*rs73555604*	*IFN-λ4*	*N*	*%*	*N*	*%*	N	*%*
1	G	C	T	T	C	No	926	91.9	692	68.8	386	29.2
2	G	C	—	G	C	Yes	76	7.5	177	17.6	428	32.4
3	G	C	—	G	T	Yes	—	—	17	1.7	347	26.3
4	A	C	—	G	C	Yes	5	0.5	119	11.8	99	7.5
5	G	G	—	G	C	Yes	—	—	—	—	59	4.5
Total chromosomes							1007	99.9	1005	99.9	1319	99.9

Shaded—alleles of markers that define specific haplotypes. 1000 Genomes Project populations are described in Fig. 1.

P70S is rare in East Asians (<0.5%) but has a moderate frequency (7 and 12%) in African and European ancestries, respectively. IFN-λ4 with serine at amino acid position 70 (70S, corresponds to minor rs117648444-A allele) was found to be less active compared with the more common version, IFN-λ4-70P, in different functional assays (Terczynska-Dyla and others 2014). The 2 *IFNL4* variants—rs368234815 that controls the production of IFN-λ4 and rs117648444 (P70S) that modulates its activity—together define 3 functional states of IFN-λ4 that have been associated with HCV clearance—no, weak, or strong protein (Table 3). Importantly, since there is no IFN-λ4 protein encoded by the haplotype with the rs368234815-TT allele, there is neither a 70P nor a 70S version of IFN-λ4, even in the presence of the rs117648444-G allele. Because only the rs368234815-dG allele provides functional significance to rs117648444, this marker should not be tested alone. The combination of rs368234815 and rs117648444 improved and explained the association with viral clearance and expression of interferon-stimulated genes, providing support for the primary causal role of IFN-λ4 and its variants in these associations (Galmozzi and Aghemo 2014; Terczynska-Dyla and others 2014; Bhushan and others 2017; Bhushan and Chinnaswamy 2018; Galmozzi and others 2018).

**Table 3.** Combined Effects of *IFNL4* Haplotypes on IFN-λ4 Activity and Hepatitis C Virus Clearance

			*Ex 1*					*1000 Genomes Project populations*		
	*Ex 2*		*rs368234815*							
	*P70S*	*Intr 1*	*IFN-λ4/no IFN-λ4*		*Upstream of IFNL4*	*Outcome*		*East Asian* N* = 504*	*European* N* = 503*	*African* N* = 661*
*Haplotypes*	*rs117648444, allele, aa*	*rs12979860*	*rs11322783*	*rs74597329*	*rs8099917*	*IFN-λ4*	*HCV clearance*	*%*	*%*	*%*
1	G	C	T	T	T	No	Best	91.8	68.8	29.2
2	A, 70S	T	—	G	T	Weak	Intermed	0.5	11.8	7.5
3	G, 70P	T	—	G	G	Strong	Worst	7.5	16.4	4.2
4	G, 70P	T	—	G	T	Strong	Worst	0	2.6	55.07
5	G, 70P	C	—	G	T	Strong	Worst	0	0	3.8

Shaded—haplotypes associated with HCV clearance and functional effects on IFN-λ4 activity. rs8099917-T allele captures separation between no and weak versus strong IFN-λ4 activity but only in East Asians and most Europeans. In individuals of African ancestry, rs8099917 is excluded from the minimal common associated haplotype. 1000 Genomes Project populations are described in Fig. 1.

Understanding the relationships between *IFNL4* markers also helps to explain population- and marker-specific associations detected by GWASs and other studies (Ge and others 2009; Suppiah and others 2009; Tanaka and others 2009; Thomas and others 2009; Vergara and others 2018). Specifically, in most Europeans (except for carriers of haplotype 4 with 2.6% frequency, Table 3), rs8099917 captures the combined effect of rs368234815 and rs117648444, with rs8099917-T allele representing no or weak IFN-λ4 and rs8099917-G allele representing strong IFN-λ4. In East Asians, where the contribution of rs117648444 is negligible (<0.5% frequency), rs8099917 represents the same signal as the *IFNL4* markers rs12979860 or rs368234815. However, in individuals of African ancestry, rs8099917 is not informative to capture associations with HCV clearance detectable by *IFNL4* markers.

### *IFNL4*-K154E

Recently, a detailed functional analysis was reported for a rare genetic variant within exon 5 of *IFNL4* (Bamford and others 2018). This SNP, rs377155886-A/G, corresponding to K154E (K (AAA)->E (GAA)), was not found in any of the 1000 Genomes Project populations (or by our sequencing of several thousand of HCV patients), but was reported in 29 of 30,896 individuals (0.047% allele frequency) with information available in dbSNP (www.ncbi.nlm.nih.gov/snp/rs377155886#frequency_tab). All the 29 individuals with the rs377155886-C allele (IFN-λ4-154E protein variant) were from African populations. Although this allele is very rare and does not contribute to the genetic association with HCV clearance detected for the GWAS markers, it has some interesting functional properties. Specifically, all mammals that are predicted to generate IFN-λ4 invariably carry the ancestral rs377155886-C allele that encodes IFN-λ4-154E protein (Paquin and others 2016; Bamford and others 2018), but this ancestral allele is found only in the 29 African individuals reported in dbSNP. The presence of the derived 154K allele strongly decreased secretion and functional activity of IFN-λ4 (Bamford and others 2018). The unusual near-complete replacement of the ancestral 154E allele by the derived human-specific 154K allele suggests that restriction of IFN-λ4 activity was extremely beneficial and caused quick fixation of the 154K allele in humans, but the mechanisms of this extreme selection are unclear.

### *IFNL3* variants in high LD with rs12979860

The candidate LD block includes 3 *IFNL3* SNPs linked with rs12979860 (Table 2). SNP rs28416813 is located within the 5′UTR of *IFNL3* and might be functionally relevant for transcriptional regulation of *IFNL3*. Functional significance of rs688187, located downstream of *IFNL3*, is unknown, but this SNP was reported to be associated with mucinous ovarian carcinoma in a GWAS (Kelemen and others 2015), likely as a proxy for other variants in this LD block.

SNP rs4803217 is located in the 3′UTR of *IFNL3* and has been reported to affect *IFNL3* mRNA stability (McFarland and others 2014; Lu and others 2015b). Based on the functional activity of rs4803217, it has been discussed whether this marker and not the IFN-λ4-controlling rs368234815 is a primary variant responsible for genetic and functional association with HCV clearance in this region. Because rs4803217 is included in the candidate LD block in all populations (Table 1), there is little power to resolve this question purely based on statistical analysis, at least in East Asians and Europeans, where these markers are in a near-complete LD.

One such effort was done in African American HCV patients where lower *r*^2^ (correlation) between these markers affords more power to address this question (O'Brien and others 2015). In a single marker analysis, the association with spontaneous and treatment-induced HCV clearance was significantly stronger for rs368234815-dG than for rs4803217-T allele. A 2-marker haplotype analysis provided additional information. The haplotype with rs4803217-T allele (linked with decreased *IFNL3* stability) and the IFN-λ4-producing rs368234815-dG allele was found to be associated with decreased HCV clearance, but because rs4803217-T always segregates with rs368234815-dG, it is hard to determine which of these alleles is functional. However, a minor haplotype that included the unfavorable rs368234815-dG allele and the favorable rs4803217-G allele (linked with higher *IFNL3* stability) was associated with significantly worse viral clearance compared with any other combination of these markers (O'Brien and others 2015). These results indicate that higher *IFNL3* stability is either irrelevant or not as favorable for HCV clearance as it thought to be, or that rs368234815 and rs4803217 might have functional effects in different conditions. Owing to a more restricted expression pattern of IFN-λ4 compared with IFN-λ3, rs4803217 effect on *IFNL3* stability may be significant enough in those conditions when IFN-λ4 is not expressed. However, when IFN-λ4 is expressed, this might have a stronger functional effect than the variability in *IFNL3* stability, making rs368234815 the main functional variant in this region.

### Is there IFN-λ5 encoded by the *IFNL* locus?

**FIG. 2.**
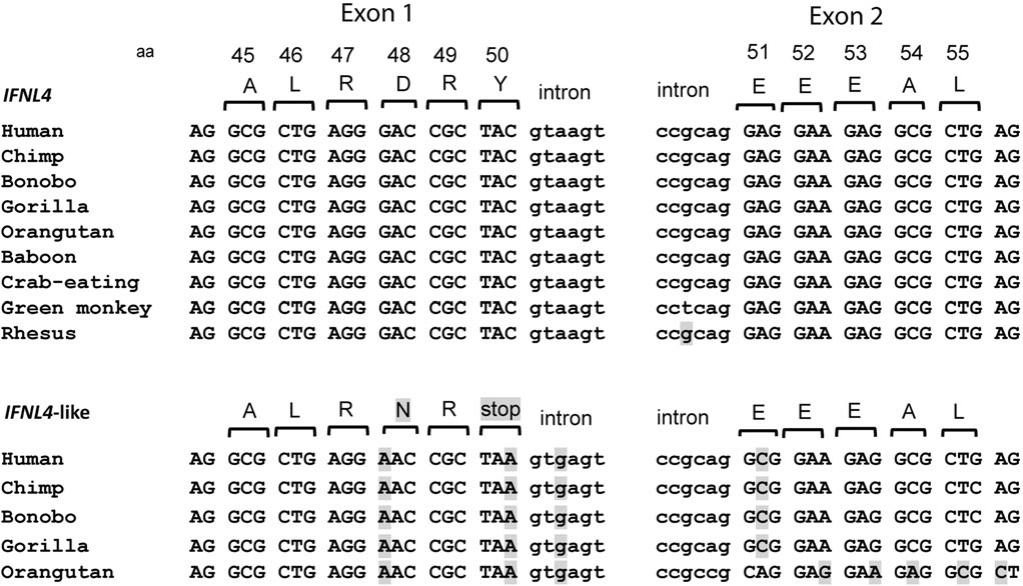
Analysis of sequences from the *IFNL4* and *IFNL4*-like regions. *Shaded*-nucleotides and amino acids that differ from the human *IFNL4* sequence. Sequences from several species were retrieved from UCSC Browser and annotated using ClustalW alignment (http://www.ebi.ac.uk/Tools/msa/clustalo). Amino acid Y50 amino acid (TAC codon) is encoded by exon 1 of *IFNL4 (*upstream of *IFNL3),* whereas a similar sequence of the *IFNL4*-like region (upstream of *IFNL2*) encodes a stop codon (TAA), resulting in an invariable truncation of the putative IFN-λ4 protein after 49 aa. Sequences of the *IFNL4*-like region in other primates are more diverse and unlikely to encode any IFN-λ4-like protein fragments.

**FIG. 3.**
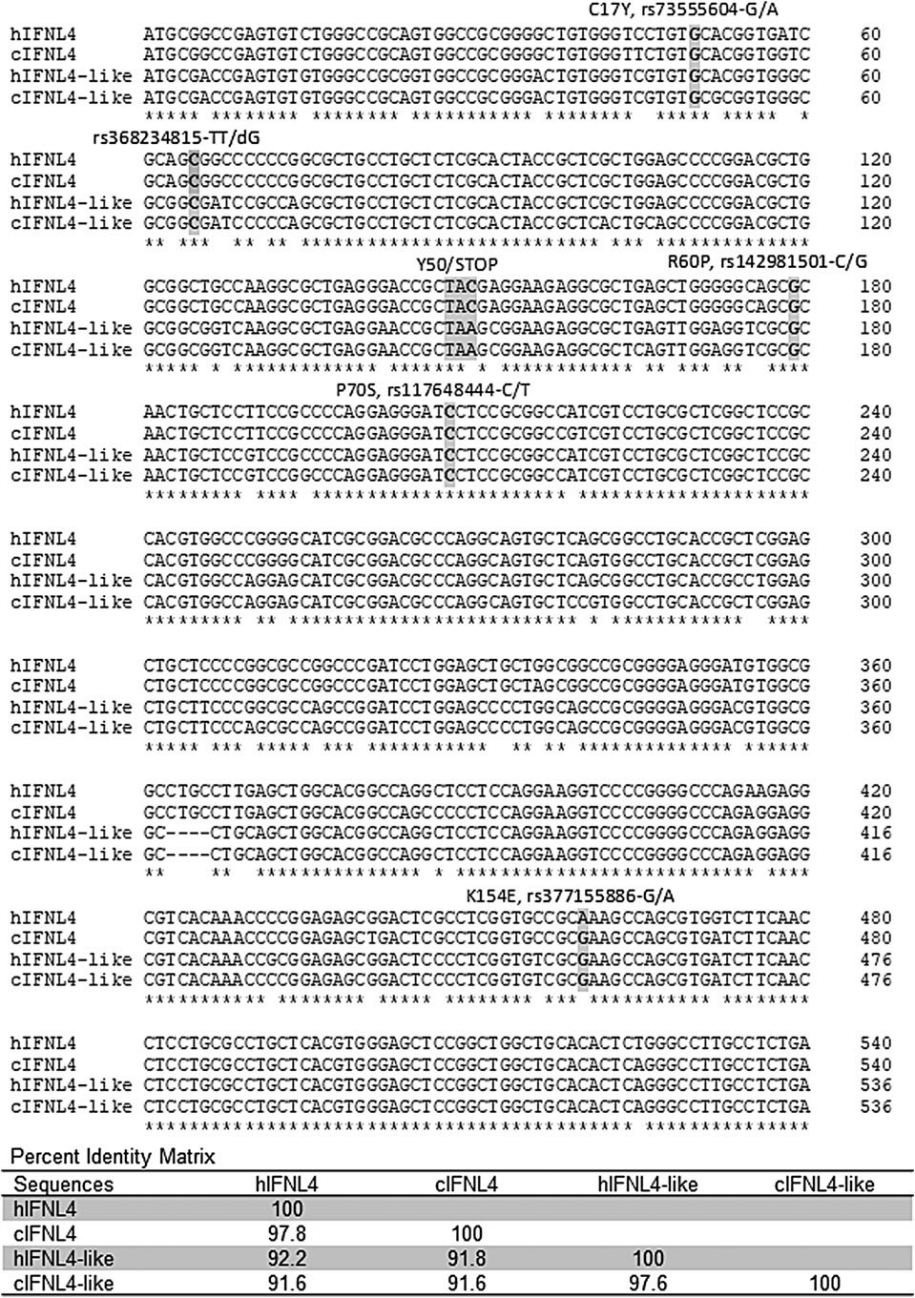
Clustal W alignment of *IFNL4* and *IFNL4*-like DNA sequences from human (h) and chimpanzee (c) genomes. *Shaded*—the location of protein-coding genetic variants discussed in the text. Y50/STOP position represents the critical difference between *IFNL4* and *IFNL4*-like regions. A percentage identity matrix is based on ClustalW alignment. Except for K154E, major alleles of all genetic variants are ancestral alleles present both in human and chimpanzee *IFNL4* and *IFNL4*-like regions. For K154K, the human-derived allele rs377155886-A is a currently nearly fixed allele with 99.01% frequency.

Because *IFNL2* and *IFNL3* genes are located within highly conserved duplicated regions and *IFNL4* is located upstream of *IFNL3,* there was always a question of whether the region upstream of *IFNL2* might harbor yet another *IFNL4*-like gene? In fact, analysis using the full-length coding sequence of human *IFNL4* (540 bp) shows a highly similar sequence upstream of *IFNL2*, with 92.2% of sequence identity with *IFNL4*. Despite this high similarity, only a short 49 aa protein fragment can be produced from this transcript, with an early and invariable termination at the end of the first exon (Prokunina-Olsson and others 2013) (Fig. 2). The stop codon is also present within *IFNL4*-like sequences of several other Hominidae species tested—chimpanzee, gorilla, bonobo, and orangutan (Fig. 2). In other primates, this *IFNL4*-like region either does not exist or is more diverse and could not be properly aligned. Thus, great apes have 2 highly similar regions (91.6% identity in chimpanzee, Fig. 3), with only 1 of these regions producing full-length IFN-λ4 protein. It is possible that the invariable stop codon in the *IFNL4*-like region was acquired after segmental duplication of the *IFNL4* region in the Hominidae lineage, as a mechanism to prevent IFN-λ4 expression. It is unknown whether the *IFNL4*-like region is expressed on the mRNA level, but this high sequence similarity should be considered when designing expression assays.

### Concluding remarks

The GWAS findings a decade ago resulted in the discovery of a novel human interferon, IFN-λ4, in 2013 and reinvigorated interest to other type-III interferons (IFN-λ1, 2, and 3), discovered in 2002. Genetic regulation of functional properties of IFN-λ3 and IFN-λ4 affected by the GWAS signals remains a topic of discussions, confusions, and misinterpretations in the literature. With some *IFNL3* variants such as rs4803217 and rs28416813, possibly functionally affecting the quantity or quality of IFN-λ3 and *IFNL4* variants affecting the production and activity of IFN-λ4, this might be a complex complementary system that needs to be thoroughly explored both genetically and functionally.

**FIG. 4.**
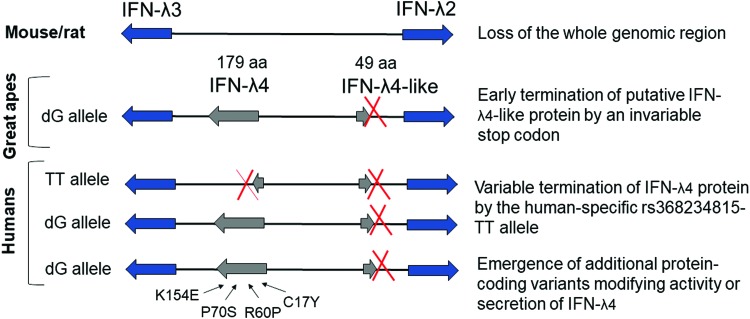
Elimination or modulation of IFN-λ4 activity by several evolutionary mechanisms. The whole region encoding IFN-λ4 protein is absent in the mouse and rat genomes. In great apes, the *IFNL4*-like sequence upstream of *IFNL2* produces only a 49 aa protein fragment due to an invariable stop codon (Fig. 2). A derived human-specific allele rs368234815-TT eliminates the open reading frame for IFN-λ4 in ∼50% of the world population by introducing a stop codon resulting in the production of aberrant non-IFN-λ4 protein fragments of 51, 75, 123, 124, or 143 aa, depending on the alternative exons used. Additional protein-coding variants emerged on the background of the IFN-λ4-producing haplotype with dG allele. The effects of P70S and K154E variants have been related to the decrease of IFN-λ4 activity or/and secretion, whereas functional effects of Y17C and R60P remain to be explored. Color images are available online.

The important role of the *IFNL* region is suggested by several partially redundant mechanisms, all resulting in restriction of IFN-λ4 activity—through elimination of the whole region (in mice and rats), introduction of a stop codon in the *IFNL4*-like region in the Hominidae lineage, and, eventually, by introduction of human-specific genetic variants within *IFNL4* eliminating or modulating IFN-λ4 activity (Fig. 4). The exact biological activity of IFN-λ4 that needs to be safeguarded and modulated by so many mechanisms remains poorly understood but is being actively investigated.

Starting from the initial GWAS findings for HCV clearance, the representative *IFNL4* markers have now been associated with multiple conditions, such as postpartum adaptive immune response (Honegger and others 2016; Price and others 2016), altered immune cell population profiles in peripheral blood (O'Connor and others 2016), outcomes of transplantation (Manuel and others 2015; Corrales and others 2017), AIDS-related Kaposi's sarcoma (Bibert and others 2018), liver inflammation and fibrosis (Eslam and others 2017), and damage in nonalcoholic fatty liver disease (Petta and others 2017). In addition to association with mucinous ovarian cancer (Kelemen and others 2015), another cancer connection was recently established for the risk of aggressive prostate cancer in men exposed to sexually transmitted infections (Minas and others 2018) and higher risk of developing interferon signature in prostate tumors and decreased survival of prostate cancer patients (Tang and others 2018). Response to interferon alpha used for the treatment of myeloproliferative neoplasm was also associated with *IFNL4* markers (Lindgren and others 2018). It is remarkable that all these findings became possible only because of GWAS discoveries made 10 years ago and all the genetic and functional studies they have ignited.
